# Triple Burden of Neurocognitive Impairment and Co-occurring Depression and Anxiety Among People Living With HIV in Bahir Dar, Ethiopia: A Multicenter Study

**DOI:** 10.3389/fpsyt.2022.732229

**Published:** 2022-04-26

**Authors:** Minale Tareke, Tilahun Belete, Temesgen Ergetie, Meseret Tadesse, Melak Menberu, Asmamaw Ketemaw

**Affiliations:** ^1^Department of Psychiatry, College of Medicine and Health Science, Bahir Dar University, Bahir Dar, Ethiopia; ^2^School of Public Health, College of Medicine and Health Science, Bahir Dar University, Bahir Dar, Ethiopia

**Keywords:** neurocognitive impairment, people living with HIV, co-occurring, depression, anxiety

## Abstract

**Background:**

Neurocognitive impairment is associated with psychological morbidities, such as depression and anxiety, among people living with HIV. The presence of these comorbidities affects viral load suppression, treatment adherence, quality of life, treatment outcomes, and functionality. Despite this fact, there is a dearth of studies that examined the triple burden of neurocognitive impairment and co-occurring depression and anxiety among antiretroviral therapy attendees in Ethiopia. This study aimed to assess the magnitude of HIV-associated neurocognitive impairment and co-occurring depression and anxiety at the same time among people living with HIV/AIDS.

**Method:**

We conducted an institution-based multicenter cross-sectional study in Bahir Dar, Northwest Ethiopia. A total of 410 study participants were selected using a systematic random sampling technique. Neurocognitive impairment was assessed using the International HIV Dementia Scale. Co-occurring depression and anxiety were assessed using the Hospital Anxiety and Depression Scale. A semi-structured questionnaire was applied to collect data on sociodemographic and clinical-related characteristics. Data were analyzed using descriptive statistics and univariate and multivariable logistic regression.

**Results:**

Two-thirds (66.8%) of the people living with HIV had neurocognitive impairment. The prevalence of co-occurring depression and anxiety was found in 39.8%. Women with HIV, people with comorbid chronic medical illness, and those under a second-line treatment regimen were factors associated with neurocognitive impairment. Furthermore, pill burden, second-line treatment regimen, HIV clinical stages, social support, HIV-perceived stigma, and neurocognitive impairment were associated factors with co-occurring depression and anxiety.

**Conclusions:**

We found a high prevalence of neurocognitive impairment and co-occurring depression and anxiety among people living with HIV/AIDs. Further research is needed to assess the clinical course of neurocognitive impairment and co-occurring depression and anxiety.

## Introduction

Despite the decrement in the prevalence of human immunodeficiency virus (HIV), the Sub-Saharan region still accounts for 59% of new HIV infections globally according to the Joint United Nations Program on HIV/AIDS (UNAIDS). In the same report, of among the 38 million people living with HIV, about 21 million (55%) live in Eastern and Southern Africa (1).

Ethiopia is one of the Sub-Saharan nations that have been suffering from HIV/AIDS. In 2019, there were 12,000 AIDS-related deaths and 15,000 new HIV infections, and the total number of people living with HIV was about 670,000. The estimated prevalence had a slight decrement from 1.4% (1–1.8%) in 2010 to.9% (0.7–1.2%) in 2019 (1–3).

Neurocognitive impairment is described as a deficit in cognitive abilities that include learning, attention, concentration, memory, executive function, decision-making skills, and problem-solving ability (4). Because of HIV infection, the Frascati criteria described three categories of HIV-associated neurocognitive disorder (HAND): asymptomatic neurocognitive impairment, mild neurocognitive disorder, and HIV-associated dementia (5).

The HIV-associated neurocognitive disorder (HAND) remains a major problem that lowers the quality of life and increases the public health burden among people living with HIV (6). Currently, the wide use of highly active antiretroviral treatment has decreased the prevalence, in particular, of the severe form of HIV-associated dementia (7).

People living with HIV experience higher rates of mental health disorders, particularly depression and anxiety, than the general population (8). Depression is characterized as the persistence of low mood associated with changes in appetite (weight), sleep pattern, and motor activity. In addition, poor concentrations, excessive tiredness, and a feeling of worthlessness that can even lead to self-harm are accounted for depression. Anxiety is a disorder when a person is unable to control his/her excessive worries, fears, or panic state, and when these symptoms are experienced frequently (9, 10).

HIV/AIDS and mental health have a direct relationship. People living with HIV can develop a mental illness at a higher rate or *vice versa* (4). For instance, people living with mental illness are at higher risk for HIV (11) due to low access to information, injectable drugs use, having multiple sexual partners, sexual abuse, and unprotected sex (12). Also, the presence of such psychiatric disorders negatively affects treatment adherence, viral load suppression, quality of life, treatment outcomes, and functionality of people living with HIV (13). The worst outcome of comorbid psychiatric disorders is significantly associated with excess mortality among adults (14).

Neurocognitive impairment associated with psychological morbidity (anxiety or depression) among people living with HIV poses complications, worse quality of life, and mortality (15). Therefore, it is very important to undertake a study focusing on neurocognitive impairment, depression, and anxiety among people living with HIV. The main objective of this study was to determine the proportions of neurocognitive impairment and co-occurring depression and anxiety, as well as its associated factors among people living with HIV. It is also in line with the global AIDS strategy (2021–2026) set by the UNAIDS to break down barriers to HIV outcomes. In addition, it can help to plan different intervention packages for people living with HIV.

## Methods

### Study Design and Period

An institution-based multicenter cross-sectional study was conducted from March to April 2016.

### Study Area and Population

This study was conducted in public health institutions in Bahir Dar. Bahir Dar is the capital city of the Amhara regional state, which is located in Northwest Ethiopia and is around 490 km from Addis Ababa, the capital city of Ethiopia. According to the 2007 Census conducted by the central statistical agency of Ethiopia, Bahir Dar Special Zone had a total population of 221,991, of whom 108,456 were men and 113,535 were women. From a total of 8,293,734 screened people from the years 2015 to 2018 in the Amhara region, 57,293 new HIV infections were reported, with an overall incidence rate of 6.9 per 1,000 population, which is the highest. The second highest incidence rate was reported in Bahir Dar (4.27 per 1,000 population) next to Dessie Town (5.74 per 1,000 population) in the Amhara region (16).

During the study period, there were two public hospitals, ten health centers (HCs), and many other private health institutions (clinics, hospitals, and pharmacies). The study was carried out in all public health institutions that provide antiretroviral therapy (ART) services in the city.

Patients with HIV/AIDS who have been on ART follow-up in public institutions in Bahir Dar city were the source population. The study population was composed of people living with HIV who had been attending their treatment in public health institutions in Bahir Dar during the study period.

### Inclusion and Exclusion Criteria

All people aged between 18 and 64 years who had follow-up for ART medication were eligible. Patients with severe medical and psychiatric (impaired insight) illnesses at the time of data collection, intellectual disability, and upper limb amputation or defects were excluded from the study.

### Sample Size and Sampling Procedures

We used the single population proportion formula *n* = (zα/2)2 p (1-p)/d2) (17) to determine the sample size with the following assumptions: 95% confidence interval (CI) (Zα/2 = 1.96), 5% margin of error, 36.4% proportion of HIV-associated neurocognitive impairment among HIV/AIDS patients from the previous study (18) and 15% contingency for non-response rate. Based on these assumptions, 410 study participants were selected for the study.

The study participants were selected using a systematic random sampling technique. For each health institution, a proportional allocation was carried out to get the demanded sample size based on the number of people living with HIV/AIDS who attended each health institution. Finally, the required sample in each health institution was enrolled through an exit interview.

### Data Collection Tools and Procedures

Data were collected using a semi-structured questionnaire that consisted of sociodemographic characteristics, clinical factors, and psychosocial variables. HIV stages stated by the World Health Organization (WHO), CD4 counts, co-morbid chronic medical diseases, total daily pill burden, and treatment regimen were collected by reviewing patients' charts.

The International HIV Dementia Scale (IHDS) was designed for use in resource-limited settings as a screening tool for HIV-associated dementia under different cultural, linguistic, and educational conditions. The scale evaluates memory, motor, and psychomotor speed without literacy-dependent tests. The maximum score of the tool is 12, with a higher score indicating better functioning (19). The IHDS is the most common HIV-associated neurocognitive disorder (HAND) screening tool used in many African countries including South Africa, Uganda, and Cameroon (20–23). In addition, most recent systematic reviews and meta-analyses show that the IHDS has fair diagnostic accuracy in all forms of HAND (asymptomatic neurocognitive disorder, symptomatic neurocognitive, and HIV-associated dementia) (24). The presence of neurocognitive impairment was explained by the sum of a 3-item IHDS (timed finger tapping test, alternating hand sequence test, and memory-recall test) cutoff score of ≤ 10 in different studies (19, 25). Therefore, this study used the same cutoff score to screen for neurocognitive impairment.

The Hospital Anxiety and Depression Scale (HADS) has been extensively used as a screening tool for depression and anxiety in both community and hospital settings. The advantage of the HADS is focused on psychological symptoms (can assess symptoms of anxiety and depression). The scale comprises 14 items, each is rated from 0 to 3 according to the severity of difficulty experienced (26, 27). It can be separated into two 7-item sub-scales for depression (HAD-D) and anxiety (HAD-A). The scales used a cutoff score of ≥ 8 for both HADS-A and HADS-D (28). In addition, social support was assessed using the Oslo-3 item (29). An 11-item scale was used to assess HIV-perceived stigma (30). Non-adherence was measured using a medication adherence scale that comprises 8 items with a dichotomous patient response (yes/no), and a patient missing at least one or more items on the scale was classified under poor adherence (31, 32).

### Data Quality Management

Two days of training were given to 10 data collectors (nurses) and one supervisor (BSc nurse) on how to use tools and approach the participants. The questionnaire was translated into the local language (Amharic) and then translated back into English for consistency. To see the understandability of the questionnaire, a pre-test was conducted on 5% of the sample size 1 week before the actual data collection among people living with HIV/AIDS.

Respondents were interviewed in their local language. Besides, the supervisor, the principal investigator carried out day-to-day strict supervision during the entire period of data collection. The supervisor checked the collected data for completeness, consistency, and accuracy.

### Data Management and Analysis

The collected data were entered into a computer using Epidata 3.1 and exported to SPSS software package version 26 for analysis. For all variables, cross-checking and data cleaning were carried out by running frequencies. Relevant variables of the study participants were described using percentage, frequency, and mean. First, cross-tabulation and bivariate logistic regression were conducted for each potentially explanatory variable using crude odds ratio and 95% confidence interval (CI). Variables that satisfied *p* < 0.2 were taken for further analysis into the multivariate logistic regression model to control confounding effects. Finally, *P* < 0.05 in the multivariable analysis was considered statistically significant.

### Ethical Consideration

Ethical clearance was obtained from the Research Ethical Review Board (IRB) of Bahir Dar University, College of Medicine and Health Sciences. Then, the ethical clearance was submitted to Amhara National Regional State Health Bureau. The regional health bureau wrote permission and a supporting letter for each study site before data collection.

After a clear explanation of the purpose of the study, written informed consent was obtained from each study participant during data collection. The participants were given the right to refuse to participate as well as to withdraw at any time during the study. Refusal to participate did not result in loss of medical care provided or any other benefits. The data collectors linked participants who were screened and were found to have severe anxiety and depression to the psychiatric clinic. They maintained privacy and confidentiality throughout the study by interviewing the patients alone and using a code instead of a name.

## Results

### Sociodemographic Characteristics

Four hundred people living with HIV/AIDS were involved in the study, with a response rate of 96%. The mean age of the respondents was 36.3 ± 9.7 years. Around 58.3% of the respondents were women, and most (80.5%) of them were from urban areas. About two-thirds (63.1%) of them have educational status below secondary school (Table 1).

**Table 1:** Distribution of participants by socio-demographic characteristics of the government health institution in Bahir Dar, 2016.

**Variable**		**Frequency**	**Percentage**
		**(*n* = 400)**	**(%)**
Sex	Male	167	41.8
	Female	233	58.3
Age	18–29	105	26.3
	30–39	162	40.5
	40–49	90	22.5
	50–64	43	10.8
Marital status	Married	214	53.5
	Single	95	23.8
	Divorced/Widowed	91	22.8
Residence	Urban	322	80.5
	Rural	78	19.5
Religion	Orthodox	340	85
	Muslim	53	13.3
	Protestant	7	1.8
Ethnicity	Amhara	353	88.3
	Agew	25	6.3
	Tigre	14	3.5
	Others	8	2
Educational status	Unable to write and read	135	33.8
	Primary school	117	29.3
	Secondary school	103	25.8
	Diploma and above	45	11.3
Occupation	Government employee	64	16
	Merchant	111	27.8
	Farmer	48	12
	Housewives	38	9.5
	Private employee	64	16
	No Job	75	18.8
Monthly income (Ethiopian birr)	<700	122	30.5
	700–969	77	19.3
	970–1,900	104	26
	>1,900	97	24.3

### Clinical and Psychosocial Characteristics

The median duration of illness since the first HIV test was 5 years [interquartile range (IQR) 3–8 years], while the median duration of antiretroviral therapy follow-up was 4 years (IQR 2–7 years). Almost one-fifth (18.3%) of the participants had a comorbid medical illness at the time of data collection: diabetes mellitus (8.3%), tuberculosis (6.3%), epilepsy (1.3%), and other chronic medical diseases (5.5%). Based on the most recent CD4 counts from the chart review, greater than half (56.8%) of the participants had CD4 counts of 500 cells/μl or less; among them, 69.5% had opportunistic infections (Table 2).

**Table 2:** Distribution of PLH by their clinical status at government health institution in Bahir Dar, Ethiopia, 2016.

**Variable**		**Frequency**	**Percent**
**name**		**(*n* = 400)**	
Recent CD4^*^ count (cells**/**μl**)**	<500 cells/μl	220	55
Duration of HIV status	≥500 cells/μl	180	45
known	<5 years	220	55
	5–10 years	148	37
	>10 years	32	8
Duration on HAART^*^	<5 years	207	51.7
	5–10years	177	44.3
	>10 years	16	4
Total daily pill burden	2-Jan	228	57
	>2	172	43
WHO HIV clinical staging	Stage I	154	38.5
	Stage II	159	39.8
	Stage III/IV	87	21.8
opportunistic infections	Yes	105	26.3
	No	295	73.8
Treatment regimen	First-line	325	81.3
	Second line	75	18.8
Comorbid medical diseases	Yes	73	18.3
Social Support	No	327	81.8
	Poor	84	21
	Medium	232	58
Adherence to HAAR	Strong	84	21
	Poor	70	17.5
Perceived stigma	Good	330	82.5
	Yes	185	46.3
	No	215	53.8

* *HAART- highly active antiretroviral therapy*.

*Cluster of differentiation 4 (CD4) levels*.

### HIV-Associated Neurocognitive Impairment and Co-occurring Anxiety and Depression

Two-thirds (66.8%, 95% CI: 62–71.5%) of the people living with HIV had HIV-associated neurocognitive impairment. The first assessment with the IHDS was a timed finger tapping test, and almost one–third (30.5%) of them scored 4 out of 4. On the other hand, the alternating hand sequence test (psychomotor speed) was assessed, and almost one-fifth (22%) of them scored 4 out of 4. Finally, registration of new things (memory recall) was assessed, and 16.3% of them had recalled all four items without any clue and scored 4 out of 4 (Table 3).

**Table 3:** The International HIV Dementia Scale (IHDS) Distribution of PLHA at government health institution in Bahir Dar, 2016.

**IHDS**	**Response**	**Frequency (n)**	**Percent (%)**
Motor speed	0–2 in 5 s	15	3.8
	3–6 in 5 s	37	9.3
	7–10 in 5 s	92	23
	11–14 in 5 s	134	33.5
	15 in 5 s	122	30.5
Psychomotor speed	unable to perform	16	4
	1 sequence in 10 s	41	10.3
	2 sequences in 10 s	97	24.3
	3 sequences in 10 s	158	39.5
	4 sequences in 10 s	88	22
Memory recall	1	18	4.5
	1.5	15	3.8
	*2*	46	11.6
	2.5	66	16.5
	3	93	23.3
	3.5	97	24.3
	4	65	16.3

**Figure 1:**
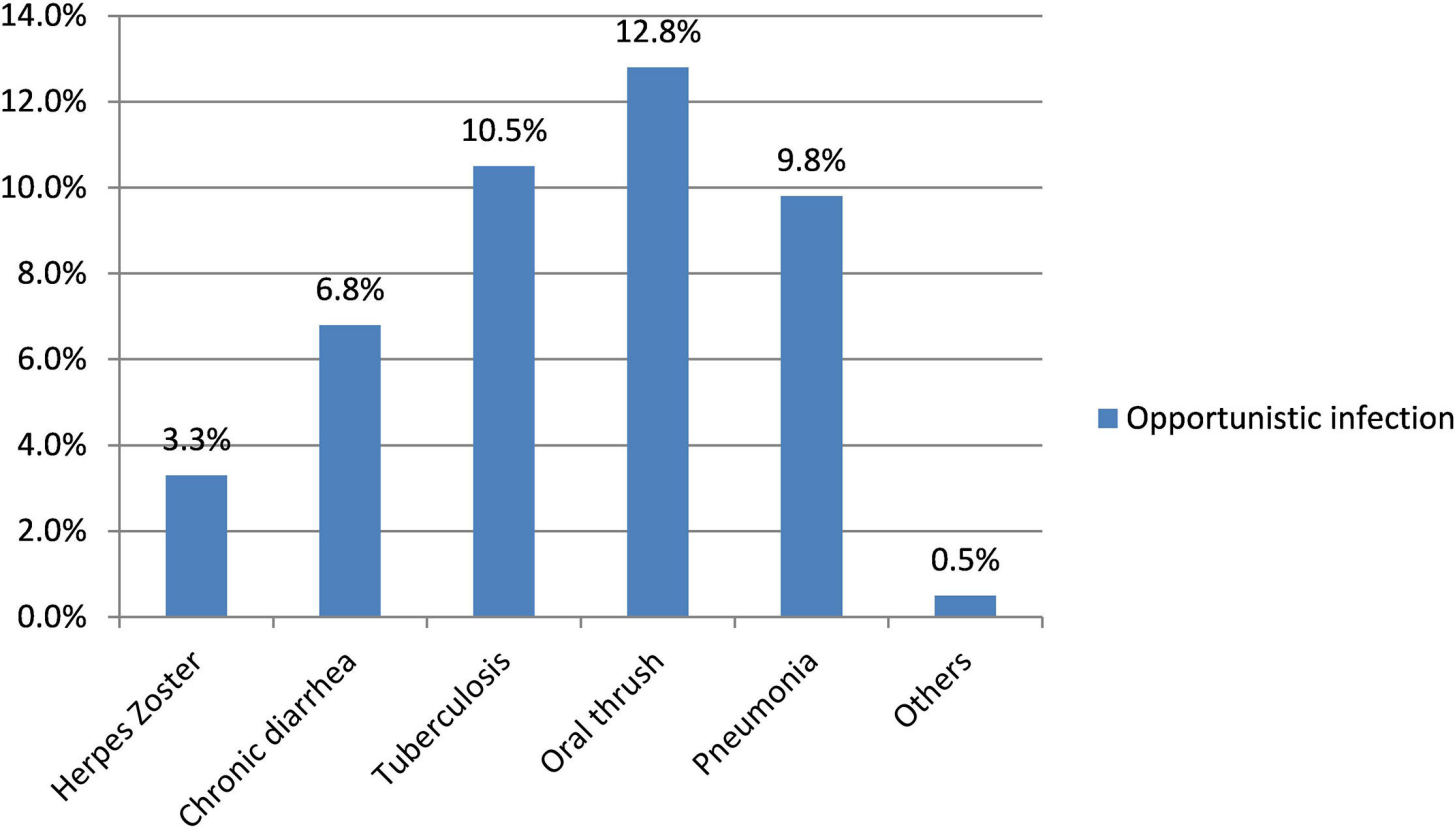
Depression, anxiety, co-occuring anxiety and depression based on sex of the respondents among PLHA at government health institution in Bihar Dar, 2016.

The magnitude of co-occurring anxiety and depression among people living with HIV/AIDS in this study was 39.8% (95% CI: 34.8–44.5). The magnitude of anxiety and depression was 58.5 and 48.8%, respectively. The magnitude of depression (55.4%) and co-occurring anxiety and depression (55.3%) was higher among the female respondents (Figure 1).

Women, people having comorbid chronic medical diseases, and people under second-line treatment regimens were around two times more likely to have HIV-associated neurocognitive impairment (Table 4).

**Table 4:** Bivariate and multivariate analysis of variables associated with NCI among PLH attending at public health institution in Bahir Dar, Ethiopia, 2016 (*n* = 400).

		**Neurocognitive impairment**				
**Variable**		**Yes**	**No**	**COR (95%CI)**	**AOR (95%CI)**	***P*-value**
Sex of respondent	Female	168	65	0.56 (0.37,0.85)	1.92 (1.24,2.97)^*^	0.01
HIV Clinical stage	Male	99	68	1	1	
	Stage I	98	56	1		
Opportunistic infection	Stage II	14	55	0.92 (0.58,1.47)		
	Stage III/IV	65	22	0.59 (0.33,1.06)		
	Yes	79	26	0.57 (0.35,0.95)		
	No	188	107	1		
Co- morbid medical diseases	Yes	60	13	0.37 (0.19,0.70)	2.28 (1.17,4.44)^*^	<0.01
	No	207	120	1		
Treatment regimen	First-line	208	117	1	1	
Social support	Second-line	59	16	2.07 (1.14,3.76)	1.94 (1.03,3.63)^*^	0.04
	poor	58	26	0.51 (0.27,0.97)		
	medium	164	68	0.47 (0.28,0.80)		
	strong	45	39	1		

* *P < 0.05*.

People living with HIV/AIDS, who have co-occurring anxiety and depression, were around two times more likely to have perceived stigma, HIV-associated neurocognitive impairment, poor social support, and a second-line treatment regimen. Also, people living with HIV with co-occurring depression and anxiety were three times more likely to be in stage III/IV. Besides, patients who have been taking greater than two pills/day were 57% less likely to develop co-occurring anxiety and depression as compared to patients who were taking less than or equal to two pills per day (Table 5).

**Table 5:** Bivariate and Multivariate analysis of variables associated with co-occurrence of depression and anxiety among PLH attending at public health institution in Bahir Dar, Ethiopia, 2016 (*n* = 400).

**Variable**		**Co-occurring depression andanxiety**				
		**Yes**	**No**	**COR (95% CI)**	**AOR (95% CI)**	***P*-value**
Recent CD4 count (cells/μl)	<500 cells/μl	98	129	1.39 (0.92,2.09)		
Total daily pill burden	≥ 500 cells/μl	61	112	1		
HIV Clinical stage	0–2 pills	106	122	1	1	
	>2 pills	53	119	0.51 (0.33,0.77)	0.43 (0.27,0.69)^*^	<0.01
	Stage I	42	112	1	1	
Opportunistic infection	Stage II	73	86	2.26 (1.41,3.63)	3.11 (1.83,5.30)^*^	<0.01
	Stage III/IV	44	43	2.73 (1.57,4.73)	3.45 (1.82,6.54)^*^	<0.01
	Yes	48	57	1.39 (0.89,2.19)		
	No	111	148	1		
						
Co- morbid medical diseases	Yes	39	34	0.50 (0.30,0.84)		
	No	120	207	1		
						
Treatment regimen	First-line	118	207	1	1	
	Second-line	41	34	2.11 (1.27,3.51)	1.87 (1.06,3.29)^*^	<0.01
Adherence to HAAR	Good	119	211	1		
Social support	Poor	40	30	2.36 (1.40,3.99)		
	poor	47	37	2.99 (1.58,5.66)	2.39 (1.20,4.74)^*^	0.01
	medium	87	145	1.42 (0.82,2.42)	1.14 (0.64,2.05)	
Perceived stigma	strong	25	59	1	1	
	Yes	71	144	1.84 (1.22,2.76)	2.09 (1.31,3.33)^*^	<0.01
Neurocognitive impairment	No	88	97	1	1	
	Yes	122	145	0.46 (0.29,0.72)	1.79 (1.10,2.92)^*^	0.02
	No	37	96	1	1	

* *P < 0.05*.

## Discussion

Among people living with HIV/AIDS who have been screened for neurocognitive impairment, 66.8% (95% CI: 62–71.5) had a neurocognitive impairment, whereas co-occurring anxiety and depression in this study was found in 39.8% (95% CI: 34.8–44.5) of the patients. The prevalence of clinically elevated symptoms of anxiety and depression was 58.5 and 48.8%, respectively.

Women with HIV/AIDS, people having a comorbid chronic medical illness, and those under a second-line treatment regimen were factors associated with neurocognitive impairment. Furthermore, pill burden, second-line treatment regimen, HIV clinical stages, social support, HIV-perceived stigma, and neurocognitive impairment were factors associated with co-occurring depression and anxiety.

The magnitude of neurocognitive impairment in the current study is consistent with the findings done in the semi-urban district of Entebbe, Uganda (64.4%) (33), Kenya (65%) (34), and Southern Ethiopia (67.1%) (35). This study has shown a much higher magnitude of neurocognitive impairment than previous studies conducted in Ethiopia (33.3–39.3%) (18, 36–38). The lower prevalence in previous studies might be due to differences in inclusion criteria, sample size variation, and instrument cutoff point differences for screening neurocognitive impairment. This study focused on the prevalence of neurocognitive impairment among people living with HIV and among those who have been taking combination antiretroviral therapy (cART), unlike others that consume both pre-and on cART medications. Furthermore, we used a large sample size (400) compared to previous studies (244 and 254), which could account for the differences. This study found that a little over half (56.8%) of the people living with HIV had a CD4 count of < 500 cells/μl. In contrast, 44% of residents of Mizan-Aman in Ethiopia who were living with HIV had a CD4 count of < 500 cells/μl during the period of data collection. Low-level CD4 count was strongly associated with severe immunosuppression and increased the prevalence as well as the severity of neurocognitive impairment (37, 39, 40).

This study reported lower neurocognitive impairment than the study done in São Paulo, Brazil (73.6%) (41), Switzerland (84%) (42), at Kenyatta National Hospital in Nairobi (88%) (43), Moi Teaching and Referral Hospital (81.1%) (44), Kenya, and at the Bamenda Regional Hospital AIDS-treatment center, Cameroon (85%) (45). This might be due to differences in sociodemographic and clinical characteristics. For instance, our study participants were somewhat younger (mean age 36.3 years) than the Brazilian (mean age 45.3 years), Kenyan (mean age 42 years), and Cameroonian (41 years) study participants. Older age is among demographic factors that are associated with reduced neurocognitive functioning and increased risk of neurocognitive impairment in people infected with HIV. There are synergistic mechanisms that increase the magnitude of neurocognitive impairment among older adults infected with HIV and accelerate cognitive aging (46). Besides, a Kenyan study found more than a two-fold (46.7%) increase in WHO clinical stage III/IV compared to our finding (21.8% stage III/IV). This is evidenced by late WHO clinical stage III/IV categories that were key predictors of HIV-associated neurocognitive impairment among people living with HIV/AIDS (35, 36).

On the other hand, the prevalence of co-occurring anxiety and depression in this study is much higher than in previous studies conducted in a university hospital, at the Department of Infectious and Tropical Diseases in Conakry, Guinea (8.1%) (47), in South Western Nigeria (21.9%) (48), and in Addis Ababa, Ethiopia (24.5%) (49). Also, the point prevalence of depression (48.8%) and anxiety (58.5%) is higher in this study than in studies conducted in Guinea (13.8 and 16.9%) (47), Nigeria (39.6 and 32.6%) (48), South Ethiopia (32 and 34.4%) (50), and Addis Ababa, Ethiopia (41.2 and 32.4%) (49). This might be due to differences in sociodemographic-related factors, inclusion criteria, different tools to screen anxiety and depression symptoms, and sample size.

However, the prevalence of depression is somewhat lower than in previous studies conducted among patients with HIV/AIDS in Sudan (63.1%) (51), Delhi, India (58.7%) (52), and Kumasi, Ghana (87%) (53). The possible reasons might be sociodemographic variation, inclusion criteria, use of different tools to screen depressive symptoms, and sample size variations. For example, the majority (79%) of the respondents in Ghana were women compared to this study (58.3%). Depression is more prevalent in women, which is supported by different bodies of literature (48, 49, 54).

Regarding clinical factors, co-morbid medical illnesses, daily pill burden, HIV clinical stage, treatment regimen, and neurocognitive impairment were associated with co-occurring anxiety and depression in this study. Depression is highly prevalent among individuals suffering from chronic medical diseases, which is supported by evidence (55, 56). A systematic review and meta-analysis revealed that the risk for a depressive disorder was 2–3 times greater in people with two or more chronic physical conditions than in those without any chronic physical condition. The odds of having a depressive disorder were 45% greater with each additional chronic condition (57).

Concerning pill burden, those who take more than two pills in 1 day were less likely to develop co-occurring anxiety and depression. The possible reason might be an increase in the pill burden when a health professional wants to treat different opportunistic infections. Different bodies of the literature revealed that having an opportunistic infection was a risk factor for developing depression in patients with HIV (56, 58–60). Moreover, immunosuppression (HIV clinical stage III/IV) in people living with HIV was frequently reported as a factor associated with depression (49, 61, 62).

In this study, depression was associated with HIV-associated neurocognitive impairment, which is supported by previous findings in which depression was associated with the risk of neurocognitive impairment (63, 64). Depression is commonly co-morbid with HIV infection, but the association is not clear on which factors may contribute to lower neurocognitive impairment (65).

Furthermore, psychosocial factors (HIV stigma and social support) were associated with co-occurring anxiety and depression in this study. These are supported by many previous studies (49, 50, 60, 62). A systemic review and meta-analysis revealed that poor social support had a statistically significant effect on depression among patients with HIV/AIDS. The odds of having depression among adults living with HIV who had poor social support were 31% higher than among those who had strong social support (66). In addition, people with depression who perceive their social support as poorer have worse outcomes in terms of symptoms, recovery, and social functioning (67). Perceived stigma is found commonly among people living with HIV and at risk for depression, anxiety, and many other psychosocial issues. Stigma has been related to risky sexual practices, delayed health-seeking behavior, low treatment follow-up, and, consequently, the presence of anxiety and depression (68).

The WHO HIV clinical staging, opportunistic infections, and social support were not statistically significant factors in the multivariate logistic regression for HIV-associated neurocognitive impairment. Furthermore, comorbid medical diseases, recent CD4 counts, and opportunistic infections were not statistically significant factors in the multivariate logistic regression for co-occurring anxiety and depression.

## Strengths and Limitations of the Study

To the best of our knowledge, this is the first study that has attempted to determine the magnitude of co-occurring depression and anxiety among a sample of people living with HIV, besides neurocognitive impairment in Ethiopia. This study tried to describe various factors associated with this co-occurrence among patients with HIV. The use of validated instruments in Ethiopia to screen depression and anxiety symptoms in patients with somatic diseases was another strength. Despite these strengths, there are a few limitations that should be considered when interpreting the findings. We have not used a standard measure to assess non-adherence to ART. We used a patient self-report that may underestimate or overestimate adherence due to recall bias. In addition, this study is conducted in selected health facilities in Bahir Dar, so the result may not represent all people living with HIV. Some important variables like viral load and substance use-related factors were not included. Furthermore, due to the cross-sectional nature of the study design, we were not able to find a cause-effect relationship.

## Conclusions

Our findings suggest that people living with HIV have a high burden of neurocognitive impairment in the era of potent combination antiretroviral therapy. In addition, people living with HIV struggle with co-occurring depression and anxiety symptoms. Women with HIV/AIDS, people having a comorbid chronic medical illness, and people under a second-line treatment regimen were factors associated with neurocognitive impairment. Pill burden, second-line treatment regimen, HIV clinical stages, social support, HIV-perceived stigma, and neurocognitive impairment were factors associated with co-occurring depression and anxiety. We recommend early screening and management of all people in ART clinics. Also, a future prospective study should be carried out to assess the clinical course of neurocognitive impairment and this highly co-occurring depression and anxiety.

## Data Availability Statement

The original contributions presented in the study are included in the article/supplementary material, further inquiries can be directed to the corresponding author.

## Ethics Statement

The studies involving human participants were reviewed and approved by Research Ethical Review Committee (IRB) of Bahir Dar University College of Medicine and Health Sciences. The patients/participants provided their written informed consent to participate in this study.

## Author Contributions

MT conceived the study design, collected, analyzed and interpreted the data, and drafted the manuscript for important intellectual content. TB interpreted the data and drafted the manuscript for important intellectual content. TE, MTad, MM, and AK contributed to the analysis of the results and writing of the manuscript. All authors contributed to the critical revision of the manuscript for important intellectual content and approved the final version to be published.

## Funding

This study was funded by Bahir Dar University College of Medicine and Health Sciences.

## Conflict of Interest

The authors declare that the research was conducted in the absence of any commercial or financial relationships that could be construed as a potential conflict of interest.

## Publisher's Note

All claims expressed in this article are solely those of the authors and do not necessarily represent those of their affiliated organizations, or those of the publisher, the editors and the reviewers. Any product that may be evaluated in this article, or claim that may be made by its manufacturer, is not guaranteed or endorsed by the publisher.
